# Assessment of Translocator Protein Density, as Marker of Neuroinflammation, in Major Depressive Disorder: A Pilot, Multicenter, Comparative, Controlled, Brain PET Study (INFLADEP Study)

**DOI:** 10.3389/fpsyt.2018.00326

**Published:** 2018-07-24

**Authors:** Antoine Yrondi, Bruno Aouizerate, Wissam El-Hage, Fanny Moliere, Claire Thalamas, Nicolas Delcourt, Marie Sporer, Simon Taib, Laurent Schmitt, Nicolas Arlicot, Deborah Meligne, Agnes Sommet, Anne S. Salabert, Sebastien Guillaume, Philippe Courtet, Florence Galtier, Denis Mariano-Goulart, Nicolas Menjot De Champfleur, Emmanuelle Le Bars, Thomas Desmidt, Mathieu Lemaire, Vincent Camus, Maria J. Santiago-Ribeiro, Jean P. Cottier, Philippe Fernandez, Marie Meyer, Vincent Dousset, Olivier Doumy, Didier Delhaye, Lucile Capuron, Marion Leboyer, Emmanuel Haffen, Patrice Péran, Pierre Payoux, Christophe Arbus

**Affiliations:** ^1^Service de Psychiatrie et de Psychologie Médicale de l'Adulte, Centre Expert Dépression Résistante FondaMental, CHRU de Toulouse, Hôpital Purpan, ToNIC, Toulouse NeuroImaging Center, Université de Toulouse, Inserm, UPS, Toulouse, France; ^2^Pôle de Psychiatrie Générale et Universitaire, Centre Expert Dépression Résistante FondaMental, CH Charles Perrens, UMR INRA 1286, NutriNeuro, Université de Bordeaux, Bordeaux, France; ^3^CHRU de Tours, Centre Expert Dépression Résistante FondaMental, Inserm U1253 iBrain, Inserm CIC 1415, Tours, France; ^4^Department of Emergency Psychiatry and Postacute Care, Lapeyronie Hospital, CHU Montpellier, Expert Center for Resistant Depression, Fondation Fondamental, Montpellier, France; ^5^CIC 1436, Service de Pharmacologie Clinique, CHU de Toulouse, INSERM, Université de Toulouse, UPS, Toulouse, France; ^6^Centre Anti Poison CHU Toulouse Purpan, ToNIC, Toulouse NeuroImaging Center, Université de Toulouse, Inserm, UPS, Toulouse, France; ^7^Service de Psychiatrie et de Psychologie Médicale de l'Adulte, Centre Expert Dépression Résistante FondaMental, CHRU de Toulouse, Hôpital Purpan, Toulouse, France; ^8^CHRU de Tours, Unité de Radiopharmacie, Tours, France; ^9^UMR 1253, iBrain, Université de Tours, Inserm, Tours, France; ^10^INSERM CIC 1415, University Hospital, Tours, France; ^11^Institut des handicaps des Handicaps Neurologiques, Psychiatriques et Sensoriels, FHU HoPeS, CHU Toulouse, France; ^12^Unité de Soutien Méthodologique à la Recherche Clinique (USMR), CHU de Toulouse, Toulouse, France; ^13^Departement de Médecine Nucléaire, CHU Toulouse, Toulouse, France; ^14^ToNIC, Toulouse NeuroImaging Center, Université de Toulouse, Inserm, UPS, Toulouse, France; ^15^INSERM U1061, Université de Montpellier, Montpellier, France; ^16^Centre Hospitalier Régional Universitaire Montpellier, Montpellier, France; ^17^PhyMedExp, Université de Montpellier, INSERM U1046, CNRS UMR 9214, Montpellier, France; ^18^Département de Médecine Nucléaire, CHU de Montpellier, Montpellier, France; ^19^Département de Neuroradiologie, Hôpital Gui de Chauliac, Centre Hospitalier Régional Universitaire de Montpellier, Montpellier, France; ^20^Institut d'Imagerie Fonctionnelle Humaine, Hôpital Gui de Chauliac, Centre Hospitalier Régional Universitaire de Montpellier, Montpellier, France; ^21^Laboratoire Charles Coulomb, CNRS UMR 5221, Université de Montpellier, Montpellier, France; ^22^Département d'Imagerie Médicale, Centre Hospitalier Universitaire Caremeau, Nîmes, France; ^23^CHRU de Tours, INSERM U1253, Université François Rabelais de Tours, Tours, France; ^24^Service de Médecine Nucléaire, CHRU Tours, Tours, France; ^25^Service de Neuro radiologie, CHRU Tours, Tours, France; ^26^Departement de Médecine Nucléaire, Hopital Pellegrin, Bordeaux, France; ^27^Institut de Neurosciences Cognitives et Intégratives d'Aquitaine (UMR-5287), Université de Bordeaux, Bordeaux, France; ^28^CHU Bordeaux Neurocentre Magendie, INSERM U1215, Université de Bordeaux, Bordeaux, France; ^29^Pôle de Psychiatrie Générale et Universitaire, Centre Expert Dépression Résistante FondaMental, CH Charles Perrens, Bordeaux, France; ^30^INRA, Nutrition and Integrative Neurobiology (NutriNeuro), UMR 1286, University of Bordeaux, Bordeaux, France; ^31^Pôle de Psychiatrie des Hôpitaux Universitaires, Centre Expert Dépression Résistante FondaMental, Hôpital Henri Mondor-Albert Chenevier, AP-HP, Créteil, France; ^32^INSERM U955, Translational Psychiatry, Paris-Est University, Créteil, France; ^33^Department of Clinical Psychiatry, Clinical Investigation Center 1431-INSERM, EA 481 Neurosciences, University of Bourgogne Franche-Comté, University Hospital of Besancon and FondaMental Foundation, Créteil, France

**Keywords:** depressive disorder, depression, neuroinflammation, inflammation, TSPO, DPA-714, cytokines

## Abstract

**Background:** Major depressive disorder (MDD) is a serious public health problem with high lifetime prevalence (4.4–20%) in the general population. The monoamine hypothesis is the most widespread etiological theory of MDD. Also, recent scientific data has emphasized the importance of immuno-inflammatory pathways in the pathophysiology of MDD. The lack of data on the magnitude of brain neuroinflammation in MDD is the main limitation of this inflammatory hypothesis. Our team has previously demonstrated the relevance of [18F] DPA-714 as a neuroinflammation biomarker in humans. We formulated the following hypotheses for the current study: (i) Neuroinflammation in MDD can be measured by [18F] DPA-714; (ii) its levels are associated with clinical severity; (iii) it is accompanied by anatomical and functional alterations within the frontal-subcortical circuits; (iv) it is a marker of treatment resistance.

**Methods:** Depressed patients will be recruited throughout 4 centers (Bordeaux, Montpellier, Tours, and Toulouse) of the French network from 13 expert centers for resistant depression. The patient population will be divided into 3 groups: (i) experimental group—patients with current MDD (*n* = 20), (ii) remitted depressed group—patients in remission but still being treated (*n* = 20); and, (iii) control group without any history of MDD (*n* = 20). The primary objective will be to compare PET data (i.e., distribution pattern of neuroinflammation) between the currently depressed group and the control group. Secondary objectives will be to: (i) compare neuroinflammation across groups (currently depressed group vs. remitted depressed group vs. control group); (ii) correlate neuroinflammation with clinical severity across groups; (iii) correlate neuroinflammation with MRI parameters for structural and functional integrity across groups; (iv) correlate neuroinflammation and peripheral markers of inflammation across groups.

**Discussion:** This study will assess the effects of antidepressants on neuroinflammation as well as its role in the treatment response. It will contribute to clarify the putative relationships between neuroinflammation quantified by brain neuroimaging techniques and peripheral markers of inflammation. Lastly, it is expected to open innovative and promising therapeutic perspectives based on anti-inflammatory strategies for the management of treatment-resistant forms of MDD commonly seen in clinical practice.

Clinical trial registration (reference: NCT03314155): https://www.clinicaltrials.gov/ct2/show/NCT03314155?term=neuroinflammation&cond=depression&cntry=FR&rank=1

## Introduction

Major depressive disorder (MDD) is a serious public health problem (1) with high lifetime prevalence (4.4-20%) in the general population (2). Impairment of quality of life is comparable to chronic diseases such as low back pain, high blood pressure, or cancer (3, 4). MDD is considered a “deactivation of psychosocial function” (5). It usually appears as part of a complex picture of comorbidities that are most often not or hardly managed since they may not be recognized (6). Standard therapeutic strategies often fail in up to one-third of cases in everyday clinical practice (7). The definition of treatment-resistant depression varies in the literature. Nevertheless, it is characterized by the absence of significant symptomatic remission after two successive attempts with antidepressants of different pharmacological classes, well-conducted in terms of dosage and duration, whilst ensuring quality observation (at least 80% of treatments made during the period under consideration) (8, 9).

The monoamine hypothesis is the most widespread aetiological theory of MDD (10, 11). Hence, all available antidepressants primarily act by increasing monoamine neurotransmission (serotonin, dopamine, and noradrenaline) (12). Consequently, hypofunction of the brain monoamine systems is among the most critical neurochemical mechanisms implied in MDD.

Recent scientific data has emphasized the importance of immuno-inflammatory pathways in the pathophysiology of MDD based on 4 main observations. First, a high prevalence of depressive symptoms is observed in chronically ill patients treated with the pro-inflammatory cytokine interferon-alpha (13, 14). Second, there is a high frequency of depressive symptoms in patients suffering from autoimmune or chronic inflammatory diseases (15–18). Third, a significant association has been found between an increase in the inflammatory marker CRP and the subsequent onset of depressive symptoms (19–22). Fourth, there is an increase in circulating cytokines, both at peripheral and central levels, in depressed patients (23, 24). Amongst them, TNFα and IL-6 are most often increased. The results concerning IL-1β and IL-8 are more contradictory (23). These cytokines in the central nervous system (CNS) appear to result from microglia activation (25–27). Microglia activation is one of the characteristics of neuroinflammation (28). This contributes to the secretion of pro-inflammatory cytokines and nitric oxide (NO) (29). Microglia also contributes to the resolution of inflammation by releasing anti-inflammatory factors. This theory seems to be part of a broader bio-psycho-social conceptual framework. Indeed, it has largely been documented that exposure to stressful life situations such as social aggression, traumatic events of life, etc. known to increase the risk for the further development of depressive episodes, have a significant impact on the functioning of the Immune-Hypothalamo-pituitary-adrenal axis (30, 31). In addition, epigenetic mechanisms are presumably implicated in those functional alterations related to stress (31–33).

In parallel, neuroimaging data reveal that a wide range of brain regions and networks may be involved in MDD, reflecting the complexity and diversity of depressive states (34). Relationships with the inflammation status have been established. A negative correlation was found between plasma CRP levels and the thickness of the right medial prefrontal cortex in MDD. This negative association is secondary to enhanced production of neurotoxic metabolites such as quinolinic acid from the kynurenine pathway activated in inflammatory conditions (35). Savitz et al. have also found a strong relationship with the reduction in striatum volume in a MDD sample. Furthermore, striatal volume was correlated with several depression-related items primarily referring to “concentration difficulties,” “lassitude,” and “pessimism” (36). Numerous neuroimaging studies (37–41) in MDD have found functional abnormalities of those neuronal systems in MDD in either resting conditions or during specific challenging tasks. Also, peripheral inflammation is associated with the anatomical and functional changes that occur during MDD. Corticostriatal functional connectivity is decreased in depressed patients, which is negatively correlated with plasma levels of CRP and IL-6 (42).

The lack of data on the magnitude of brain neuroinflammation in MDD is the main limitation of the inflammatory hypothesis of MDD. Positron emission tomography (PET) imaging may provide a better understanding of the pathophysiology of MDD involving cases of CNS brain inflammation. The development of new radiotracers used in PET helps us to explore and quantify neuroinflammation in depressed patients. Many of these radiotracers specifically have as target the translocation protein (TSPO). The TSPO is an 18 kDa protein located in the outer mitochondrial membrane of microglia cells. Its density is measured by its distribution volume, which was shown to be increased when microglia is activated thereby contributing to consider changes in TSPO expression as a relevant biomarker for brain neuroinflammation (43). An initial study conducted by Hannestad et al. (44) using a carbonaceous radiotracer of TSPO (11-N-(2-methoxybenzyl)-N-(4-phenoxypyridin-3-yl)acetamide-[11C] PBR28) found no significant increase in TSPO in acute-phase MDD patients (*n* = 10). However, this study was conducted in a small group of patients who were clinically heterogeneous in terms of disease severity. Setiawan et al. recently reported a significant increase in the TSPO distribution volume in 20 patients with MDD in the right and left prefrontal cortex, anterior cingulate gyrus, and insula using a fluorinated marker ([18F] FEPPA) (45). To our knowledge, these are the first data to suggest that MDD is characterized by neuroinflammation found in specific brain regions implicated in the cognitive and emotional processing of environmental stimuli (45).

Our team recently demonstrated the relevance of [18F] DPA-714 as a neuroinflammation biomarker in humans with various neurological diseases (46). The [^18^F] DPA-714 and [^18^F] FEPPA molecules are two second radiotracers targeting TSPO (overexpressed microglial receptor with inflammation). The [^18^F]DPA-714 tracer has been studied extensively in humans and also in preclinical models (46–49). This tracer was also selected by the European consortium InMind (Imaging of Neuroinflammation in Neurodegenerative Diseases−7th Framework Programme of the EU—www.uni-muenster.de/InMind) as the gold standard for PET neuroinflammation imaging with TSPO. Since [^18^F] DPA-714 appears to be the currently preferred tracer for studying neuroinflammation in PET, it will be used in this study.

The primary objective of this study is to compare the TSPO distribution volume assessed in PET (DPA-714) between patients with moderate-to-severe MDD (“currently depressed group”) treated with antidepressants considered as ineffective (and before a new treatment strategy is initiated as recommended) and normal healthy subjects without any antidepressant treatment (“control group”).

The secondary objectives are to: (i) compare the TSPO distribution volume assessed by PET among the currently depressed patients, those in clinical remission but still being treated with antidepressants (“remitted depressed group”) and normal controls; (ii) correlate the severity of depressive symptoms, suicidal risk, and central neuroinflammation intensity evaluated by PET in the currently depressed group; (iii) measure the correlation between the distribution volume of PET-assessed TSPO and a series of structural (microstructural integrity, cortical thickness, intracerebral iron content) and functional (connectivity force of the default-mode network) MRI markers in the three subject groups; (iv) measure the correlation between the distribution volume of PET-assessed TSPO and the plasma concentrations of peripheral inflammatory markers in the three subject groups.

We formulated the following hypotheses: (i) Neuroinflammation in MDD can be measured by [18F] DPA-714; (ii) its levels are associated with clinical severity; (iii) it is accompanied by anatomical and functional alterations within the frontal-subcortical circuits that are highly involved in MDD; (iv) it is a marker of treatment resistance. If these hypotheses are confirmed, then this study will enhance our understanding of the role of these neuroinflammatory processes in MDD. This breakthrough could have a significant long-term therapeutic impact by developing more specific antidepressant drugs with potent anti-inflammatory action and/or medicines primarily targeting neuroinflammation.

## Subjects and methods

### Subjects

Our study will compare data collected in patients with moderate to severe MDD (“currently depressed group”, *n* = 20) treated unsuccessfully with classical antidepressants (SSRIs, SNRIs) before the initiation of a new antidepressant treatment according to good practice recommendations, with those included in two other study groups, comprising:

- non-depressed healthy volunteers (“control group”, *n* = 20), which enables us to control both the depression factor and the treatment factor for the characterization of neuroinflammation. They will be matched on age and gender to the experimental group of currently depressed patients;
- depressed patients reaching clinical remission (“remitted depressed group”, *n* = 20) and still being treated with antidepressants, which allows us to control the depression factor. We hypothesize that the presence of an effective antidepressant treatment will contribute to demonstrate a significant decrease in neuroinflammation in those depressed patients. This group will be matched to the currently depressed group according to the class of antidepressant treatments (SSRIs, SNRIs), as well as age and gender.

### Eligibility criteria

The following inclusion criteria will be applied to all study groups: (1) men and women aged 25 to 55 years, (2) affiliated to a Social Security scheme, (3) able to understand the given instructions and information about the study design, and (4) have provided written informed consent. The currently depressed group will include patients: (1) meeting the DSM-5 criteria of MDD diagnosis, (2) experiencing significant depressive symptoms (as indicated by a score > 20 on the Montgomery and Asberg Depression Rating Scale, MADRS), (3) receiving an ineffective antidepressant treatment (a well-conducted treatment at an appropriate dosage for a minimum of 6 weeks), and (4) showing plasma antidepressant levels within the therapeutic range after at least 1 additional week of antidepressant treatment with unchanged dosage. The remitted depressed group will comprise patients: (1) meeting the DSM-5 criteria of MDD diagnosis, (2) achieving remission for at least 8 consecutive weeks according to the standard DSM-5 criteria (as indicated by a MADRS score <10), and (3) receiving antidepressant medications at a stable dosage for 1 week or more. The control group will include normal healthy subjects free from any current or past psychiatric conditions and without peripheral signs of inflammation (as indicated by circulating concentrations of hs-CRP <5 mg/L).

Patients with any of the following conditions are not eligible for both the currently depressed and remitted depressed groups: (1) woman who is pregnant (test at enrolment), parturient, or lactating, (2) persons deprived of their liberty by judicial or administrative decision, (3) persons unable to express consent, (4) subjects with an identified neurodegenerative condition or a psychiatric pathology other than MDD (bipolar disorder, chronic psychotic disorder, addictive disorder except for nicotine, obsessive-compulsive disorder, post-traumatic stress disorder), (5) subjects with a history of stroke, (6) subjects with an acute infectious pathology, or chronic or acute inflammatory diseases, (7) subjects with a history of autoimmune diseases, (8) subjects receiving long-term anti-inflammatory and immunosuppressive therapy, (9) subjects with antipsychotic treatment in the last 3 weeks prior to enrolment, (10) subjects with a prescription of diazepam, midazolam, chlordiazepoxide in the last week prior to enrolment, (11) subjects with contraindications to MRI or PET imaging, (12) subjects who refuse to be informed of an abnormality detected during MRI examination.

The exclusion criteria for the control group are: (1) a significant history of psychiatric or somatic disorders, (2) a current mental disorder, (3) an active suicidal risk, (4) an ongoing psychotropic treatment.

Depressed patients will be recruited throughout 4 centers (Bordeaux, Montpellier, Tours, and Toulouse) of the French network from 13 expert centers for resistant depression (50). The subjects of the control group will be included at clinical investigation sites working locally in close collaboration with each participating expert center.

### Clinical assessment

A psychometric assessment will be performed by trained and independent psychiatrists to assess the following items.

- Depression severity will be assessed using the French version of the MADRS (Montgomery and Asberg Depression Rating Scale) (51), a hetero-evaluation scale for depression. It is amongst the most widely used in everyday practice, comprising 10 items. The total score ranges from 0 to 60. Remission is considered to have been reached when the total MADRS score is less than 10.
- Anxiety severity will be evaluated using the French version of the BAS (Brief Scale for Anxiety) (52), a hetero-evaluation questionnaire evaluating anxiety intensity comprising 10 items: 6 for general symptoms and 4 for psychological symptoms. Each item is scored from 0 to 6 (53).
- Suicidal ideation and behavior will be examined using the French version of the C-SSRS (Columbia-Suicide Severity Rating Scale) (54). It assesses suicidal ideation for a given topic ranging from ‘wish to be dead’ to ‘active suicidal ideation with specific plan and intent.’ There are 5 closed questions on suicidal ideation, 6 questions on intensity, and 4 on suicidal behavior.

### PET assessment

The ^18^F-DPA-714 and ^18^F-FEPPA molecules are two second radiotracers that target TSPO (overexpressed microglial receptor with inflammation) (55, 56). These two tracers cross the blood-brain barrier (BBB) and have a good affinity for TSPO (57, 58). The ^18^F-DPA-714 tracer is metabolized rapidly and some metabolites secondary to oxidation can cross the BBB in small amounts (59). The FEPPA tracer is also metabolized but its hydrophile metabolites do not cross the BBB (57). The amounts of injected activity and the effective patient dose are comparable between the two tracers (47, 60–62). The ^18^F-DPA-714 tracer has been studied extensively in humans as well as in preclinical models (51 references on Medline) (46–49). There is less information on the use of FEPPA (14 references on Medline, only 8 clinical studies).

Articles on the FEPPA use in humans mention an acquisition time of 125 min, which is difficult to achieve with a large number of subjects and is very uncomfortable for patients who must be immobile during these 125 min. For FEPPA, the data analysis is performed on a kinetic model with two compartments and arterial blood samples must be collected from the patient to measure the input function of the radiotracer (62). The main advantage of using ^18^F-DPA-714 is its acquisition and analysis protocol. The radiotracer is injected (200 MBq) and then a 30-min acquisition is performed 1 h after the injection. The analysis consists of calculating a standardized uptake value (SUV). Some studies have shown that using this semi-quantitative analysis method and using a reference region results in less variability than studies using a kinetic model (49). Since ^18^F-DPA-714 appears to be the preferred tracer for studying neuroinflammation on PET, it will be used in this study.

In addition, there is known a genetic polymorphism for TSPO. This polymorphism determines the tracer′s affinity for TSPO, with some persons having high affinity sites, mixed affinity sites or lower affinity sites. The two radiotracers (^18^F-DPA-714 and ^18^F-FEPPA) are both impacted by this genetic polymorphisms (63). For feasibility reasons, the TPSO genotype of subjects cannot be an inclusion criterion; nevertheless, this parameter will be taken into account during the data analysis as a covariate, allowing us to weigh the PET imaging data based on whether the individuals have high or low affinity sites.

### Biological markers

The involvement of certain pro-inflammatory cytokines in MDD was highlighted in a meta-analysis by Dowlati et al. (64) and a recent literature review by Miller et al. (23). In these two articles, higher plasma concentrations of IL-6 and TNF-alpha were found in the MDD group than in the control groups without MDD.

The biological marker assessment will involve measuring the concentration of peripheral inflammation markers that are circulating cytokines including IL-6 and TNF-alpha, especially. For each research site, samples will be taken at the Clinical Investigation Center for all subjects.

Also, hs-CRP will be assayed on the pre-screening day as part of the usual follow-up of patients and as part of the enrolment visit for volunteers in the control group (exclusion criteria for volunteers in the control group showing hs-CRP levels > 5 mg/L indicating a significant inflammatory state). All the blood samples will be collected in the morning in order to limit the impact of circadian rhythm on the measurement of inflammatory markers.

The samples will be stored by the Clinical Investigation Centers associated with each expert center before being sent to the hospital departments involved in the determination of peripheral cytokine levels (IL-6 and TNF alpha) at the end of the study.

### Outcomes

The primary outcome will be the TSPO density assessed by the volume of cerebral distribution of the tracer [18F]DPA-714 in the currently depressed group vs. the control group.

The secondary outcomes will be: (1) the TSPO density evaluated by the brain distribution volume of the [18F]DPA-714 tracer in the currently depressed vs. remitted depressed groups, (2) the clinical severity assessed by the Montgomery and Asberg Depression Scale—MADRS), BAS (Brief Scale for Anxiety), and the Columbia-Suicide severity rating scale—CSSRS), (3) MRI imaging: (i) structural (i.e., cortical thickness to quantify cortical atrophy); (ii) diffusion (i.e., the mean diffusivity for measuring microstructural integrity); (iii) T2^*^ relaxometry (i.e., the R2^*^ for measuring intracerebral iron content), and (iv) resting-state functional MRI (i.e., default mode network connectivity strength), (4) the concentrations of biological markers of peripheral inflammation (i.e., hs-CRP, IL-6, TNF alpha).

### Risk benefit balance

No direct individual benefit can be expected for subjects participating in this study. The therapeutic modalities used in these patients will not be altered by their participation in the study or by the results. Strict compliance with the inclusion and exclusion criteria will ensure there are no foreseeable risks for persons participating in this study. The imaging modalities used (MRI and PET) do not involve any foreseeable risk when patients presenting a contraindication to one or other of these examinations are not included according to the aforementioned inclusion/exclusion criteria. The principal investigator must constantly monitor, assess, and document risks and ensure that they can be managed satisfactorily.

### Experimental procedures (Table 1)

#### Preselection

Patients in both the currently depressed and remitted depressed groups will be preselected during their medical follow-up within each participating research site. Investigators will inform the patient and answer all questions about the purpose, nature of constraints, foreseeable risks, and expected benefits of the present research. They will also specify the rights of the patient regarding research and will verify the eligibility criteria. A copy of the Information/Consent form will then be given to the patient. Healthy volunteers for the control group will be invited by phone to participate into the study by a Clinical Investigation Centre clinical research associate. A copy of the information leaflet will be sent to them, giving them sufficient time to consider their participation.

**Table 1:** Summary of INFLADEP design.

	**Preselection**	**Enrolment visit D0**	**Assessment visit D7 (± 5 days)**
Patient information	✓	✓	
Collection of informed consent		✓	
Clinical examination^1^ – Validation of eligibility criteria		✓	
Collection of blood samples^2^		✓	
MRI examination			✓
PET examination			✓
Questions about concomitant treatments		✓	✓
Search for AE/SAE		✓	✓

1 *Clinical examination: discussion with doctor, MADRS, BAS, C-SSCR, MINI surveys*.

2 *To measure CRP levels in healthy volunteers and biological markers (TNF-alpha, IL-6), TSPO phenotype in all subjects, beta-HCG for women of childbearing age*.

#### Enrolment visit

After obtaining the subject′s consent, a clinical examination will be performed to verify the eligibility criteria of the potential study participants along with the collection of psychiatric and somatic history, and current treatments. This clinical examination will include MINI (Mini International Neuropsychiatric Interview) (Appendix 5), which consists of a short, structured clinical interview that allows investigators to diagnose past and current psychiatric disorders according to the DSM (65), which is an exclusion criterion for subjects in the control group. This visit will also involve conducting psychometric assessments (MADRS, BAS, C-SSCR).

A blood sample will be taken to measure hs-CRP levels (inclusion criterion for the control group). This sample will also be used to measure IL-6 and TNF-alpha if the subject is included in the study. All the samples will be taken in the morning to take into consideration, as much as possible, the effects of circadian rhythm on circulating inflammatory biomarkers. Thereafter, an appointment will be scheduled for the assessment visit depending on the availability of the subject and the different technical platforms, at 7 ± 5 days following the inclusion visit.

#### Assessment visit

A PET scan ([18F]DPA-714) and a multimodal MRI scan will be performed. The subjects will be taken to an imaging unit for an MRI examination of around 40 min duration. As a safety measure, the MRI technician will carry out the usual safety checks needed for this type of investigation. The subjects will then be placed on a table, with their head positioned to enable brain imaging to be carried out. After placing the subject′s head in the support inside the MRI scanner, the scanning sequences will be carried out. The subjects will be able to talk to the operator at all times by intercom. Subjects must in general keep still during the MRI sequences. If an abnormality is discovered, the investigator will inform the patient during a follow-up visit.

### Statistical analyses

The Clinical Investigation Center in Toulouse contributed to the design of the study. The latter also provides methodological support to the project.

#### Study sample calculation

The main objective is to compare the central neuroinflammation measured by the TSPO distribution volume assessed by PET among both currently depressed and control groups. To our knowledge, this is the first study using the [18F] DPA-714 as biomarkers of neuroinflammation in depression with this type of study design. So, we cannot calculate the power of the study. However, few studies of this type are available in the scientific literature. There are studies using other markers of inflammation, such as [18-F]FEPPA (45). In the absence of sufficient literature data on which to base the calculation, a population of 20 patients per group seems appropriate considering the size of neuroimaging studies in the literature. In addition, 20 patients per group will allow us demonstrating, with a power of 80%, a statistically significant difference in the distribution volume between patients with a major depressive episode and controls, in the event the measured volume is similar according to the markers (based on data from the Setiawan et al. study measuring a volume of distribution of 12.3 vs. 9.3 ml/cm^3^ in the medial prefrontal cortex).

#### Analysis of PET imaging data

To evaluate the brain kinetics of [18F] DPA-714, PET images will be co-recorded on the MNI atlas with PMOD® software. To construct time-activity curves of [18F] DPA-714, the PET images will be segmented into 13 regions of interest (ROI): precuneus, anterior cingulum, posterior, frontal, temporal, parietal, occipital cingulum, hippocampus, semi-oval centrum, anterior putamen, posterior putamen, caudate nucleus, and intersections, as defined in the MNI-AAL atlas. The binding potential (BP) is the ratio of B_max_ (receptor density) to KD (dissociation constant): BP = B_max_ / KD shall be calculated. Additionally, the distribution volume ratio (DVR) will be estimated from the region of interest and a reference region using, for example, Logan graphical analysis (66) with the cerebellum as reference region for the images obtained between 0 and 90 min (46). In this study, the DVR will be calculated as the slope of the linear part of the Logan analysis using PMOD software. We will calculate the BP for the 13 ROIs mentioned above, as well as for the entire brain.

For the primary hypothesis, PET data will be analyzed by multivariate ANOVA with TSPO Volume in the 13 ROIs as the dependent variables and diagnosis and genotype as fixed factors. Main effects will be considered significant at the conventional *P* ≤ 0.05. Effects in each region, will be analyzed by univariate ANOVA.

As a secondary analysis, a MANOVA including every brain region sampled (including all cortical and subcortical regions) will be performed to assess the effect of diagnosis on TSPO Volume. A partial correlation will be used in a secondary analysis to quantitate the relationship between TSPO Volume in the primary regions of interest and severity of symptoms of MDD measured by total MADRS score.

#### Analysis of MRI data

For structural MRI analysis, image processing will be performed using FSL 4.1 (FMRIB Software Library; www.fmrib.ox.ac.uk/fsl/) and an in-house software developed in Matlab (version 6.5, The MathWorks), with procedures similar to those described earlier (67–70). Using T1 anatomical image, we will identify and segment hippocampi and amygdala. Concerning diffusion weighted imaging, a diffusion tensor imaging model will be fit at each voxel, generating mean diffusivity (MD) maps. The MD maps were then registered to brain-extracted whole-brain volumes from T1-weighted images using a full affine (correlation ratio cost function) alignment with nearest-neighbor resampling. As a result of this processing, the MD maps were corrected for head movements, and shared an identical reference space with the anatomical T1-weighted volumes. For standard volumetric analysis, we will extract volume values from each segmented structure and divided this value by the total intra-cranial volume (TIV) to normalize for participant′s brain size. For each subject, the region of interest segmentation results, coregistered MD were all superimposed onto the original T1-weighted volume. The segmented structures defined the binary masks, where the mean values of MD will be calculated for each individual.

For functional MRI analysis, image processing will be performed using SPM12 (https://www.nitrc.org/projects/spm), CONN toolbox (https://www.nitrc.org/projects/conn) and an in-house software developed in Matlab (version 6.5, The MathWorks). Individual correlation maps between the defined regions (hippocampus, amygdala, anterior cingulate gyrus, frontal cortex—these areas of interest are anatomical zones defined in known and classical stereotactic spaces in neuroimaging) and the whole brain will be determined. The coordinates of the regions are identical for each subject because the images are in an identical (MNI) space. The correlation indices will be transformed into normally distributed variables using the Fisher transformation. These maps will then be used to carry out a *t*-test, corrected for multiple comparisons, between the maps of different groups. This will allow us to determine the intensity and extent of synchronous activity in the cerebral zones functionally connected to the hippocampus, the amygdala, and the prefrontal cortex.

#### Analysis of all data

PET and MRI imaging, clinical symptomatology and biological markers. PET and MRI images will be co-recorded using a method previously used by our team (71) [e.g., 71] so that the maps of all multimodal markers have the spatial consistency needed for their statistical comparison. Firstly, for each marker map, we will perform *t*-tests to compare the groups with each other (SPSS 25.0). This will allow us to quantify the structural, functional and inflammatory alterations that characterize each group (i.e., treatment-resistant MDD vs. control, responsive MDD vs. control, treatment-resistant MDD vs. responsive MDD). Secondly, we will verify that there is no spatial collocation between the atrophy marker (MRI) and the inflammation marker (DPA714 -PET) that can induce a different interpretation of PET results. Finally, we will correlate the inflammation marker maps with the structural and functional marker maps generated with MRI. This will allow us to understand whether neuroinflammation is linked to brain changes (e.g., iron deposits, functional alteration).

To study the correlation between the severity of depressive symptoms, suicide risk and the intensity of neuroinflammation, graphic representations will be made to observe the distribution of these data in the two groups concerned. The Pearson correlation coefficient will be calculated in each instance. The same analysis strategy will be used to measure the correlation between plasma concentrations of biological markers of peripheral inflammation and the intensity of neuroinflammation in the three study groups.

### Ethics

This protocol has been approved by an independent national research ethics committee (CPP Île-de-France 6; RC31/16/8918 CPP/41-17). The protocol was also registered in the clinical trial database (NCT03314155).

## Discussion

MDD is a highly prevalent disorder associated with a considerable loss of quality of life, increased mortality rates, and substantial economic costs. Because of a lack of knowledge about this disease, many patients remain untreated. Among patients given adequate medication, only 35% will achieve clinical remission. Furthermore, the probability of recurrences increases with the number of previous depressive episodes (7, 72, 73). Greater knowledge about the pathophysiology of MDD seems therefore necessary.

The inflammatory approach is an extension of the early monoamine theory of MDD, which has remained the only explanatory model for many years (74). Indeed, in inflammatory conditions, cytokines regulate monoamine neurotransmitters, notably serotonin. The most relevant mechanism involves activation of the inducible enzyme indoleamine 2,3 dioxygenase (IDO) which degrades tryptophan (TRP) into Kynurenine (KYN). Yet, TRP is a precursor of serotonin, meaning serotonin levels will be decreased (23). Similarly, inflammation is associated with lowered dopamine function (75). In addition, the pro-inflammatory cytokines appear to stimulate the hypothalamic-pituitary-adrenal axis, as one of the major stress-responsive biological system (76, 77). There is a cascade reaction at the hypothalamic and pituitary levels with an elevation in CRH and ACTH concentrations. At the adrenal level, there is an increase in cortisol synthesis. However, high plasma cortisol levels fail to decrease efficiently the activation of the transcription factor NF-kB levels controlling the expression of pro-inflammatory genes in the CNS (23). These data seem to fit into a larger framework for the pathophysiological knowledge of MDD. Indeed, the immune-mediated mechanisms occupy a pivotal position among the neurobiological determinants of MDD. Inflammation has extensively been found to generate serious deficits in synaptic plasticity as well as profound disturbances of neurotransmitter systems, underlying the cognitive and behavioral symptoms of MDD. Indeed, preclinical models of stress-induced depression have shown how peripheral and central immune components can initiate and propagate inflammatory signals in the brain. Microglia is the primary mediator of these neuroinflammatory responses and, when exposed to stress, exhibit profound morphological and functional alterations that play a key role in the development of anxious and depressive behaviors. In addition, neuroplasticity and behavioral deficits occur when microglia is distanced from homeostatic processes and engaged toward maladaptation in response to stress (78). Nevertheless, it is important to emphasize the fact that those abnormal inflammatory processes seem to take place only a subset of depressed patients (78, 79), as attested by high peripheral levels of the inflammatory marker CRP above 3 mg/L found only in 16% of a large sample of depressed patients (79), leading to consider MDD not as the sole result of an impaired immune function. Despite these considerations, the findings supporting the inflammatory hypothesis of MDD could open up significant opportunities for medical management of those depressed patients with inflammatory profile.

Altogether, these findings should open up new opportunities for MDD medical management. Two meta-analyses (80, 81) have highlighted the anti-inflammatory role of serotonin reuptake inhibitors (SSRIs). The meta-analysis by Hannestad et al. (81) has found decreased IL6 and IL-1β serum concentrations in successfully treated patients with MDD, although these data remain contradictory. In contrast, several studies have documented that depressed patients having high baseline peripheral inflammatory cytokines (IL-6, TNF-alpha) and gene expression fails to respond adequately to SSRIs (82–84). Therefore, the use of anti-inflammatory molecules such as anti-TNFα is a new research avenue for treating MDD. Reason and colleagues have shown infliximab to be effective for treating MDD in depressed patients with a CRP greater than 5 mg/L (85). In their meta-analysis, Fond et al. (80) and Abbasi et al. (86) suggested combining a COX2 inhibitor to block the production of pro-inflammatory prostaglandins with an antidepressant for the treatment of MDD. Relationships were found between an improvement in depressive symptoms and decreasing circulating levels of IL-6. In addition, brain stimulation techniques such as electroconvulsive therapy currently proposed for the management of resistant forms of MDD appear to modulate the immunoinflammatory cascade (87).

In this context, the development of biomarkers to differentiate MDD from treatment-resistant MDD is a major challenge. Demonstrating different clinical clusters in MDD through neuroinflammation could lead to more personalized patient care. Some symptoms of depression such as anhedonia, loss of interest, and psychomotor retardation are associated with biomarkers of peripheral inflammation (88–90). However, this data mainly refers to peripheral markers of inflammation. Will we find this association with neuroinflammation in our study? In a previous study, Setiawan et al. showed that the highest TSPO distribution volume occurred in MDD patients with the most severe depressive symptoms. But they did not demonstrate any relationship between neuroinflammation and peripheral markers of inflammation. Importantly, there is no available data on the TSPO distribution volume in depressed patients in clinical remission (45). Moreover, to our knowledge, although relationships with active suicidal ideation was recently established (91), there is no scientific study supporting a link between certain symptoms of MDD such as anhedonia, loss of interest and psychomotor retardation with neuroinflammation, despite available data on peripheral inflammation markers.

We will therefore try to highlight at the level of the CNS what existing scientific data currently finds at the peripheral level to attempt to show a significant correlation between peripheral and central modifications in MDD.

## Conclusion

Our study′s first objective is to investigate the link between neuroinflammation and clinical severity, as shown earlier by Setiawan (65), and also to target the relationships with certain clinical dimensions such as anhedonia, aboulia, etc. However, the current research aims to assess the effects of antidepressants on neuroinflammation as well as its role in the treatment response. It will help to clarify the putative relationships between neuroinflammation quantified by brain neuroimaging techniques and peripheral markers of inflammation. Finally, it opens innovative and promising therapeutic avenues based on anti-inflammatory strategies for the management of treatment-resistant forms of MDD which are frequently observed in clinical practice. This translational approach is expected to markedly reduce the deleterious impact of MDD on overall functioning, quality of life and economic costs due to the disease.

## Author contributions

All authors participated in the drafting of the protocol in their field of competence. AY, BA, WE-H, and FM wrote the manuscript. All the authors have corrected the manuscript. They have all been involved in the recruitment, collection of data (clinical and neuroimaging), and/or data processing (depending on their field of expertise).

### Conflict of interest statement

The authors declare that the research was conducted in the absence of any commercial or financial relationships that could be construed as a potential conflict of interest.

## Abbreviations

[18F]DPA-714: N,N-diethyl-2-(2-(4-(2-fluoroethoxy)phenyl)-5,7-dimethylpyrazolo[1,5-α]pyrimidin-3-yl)acetamide fluoro 18
BAS: Brief Scale for Anxiety
BBB: Blood-Brain Barrier
CERRP: Centre d′Etudes et de Recherches sur les RadioPharmaceutiques
CRP: C Reactive Protein
CSSRS: Columbia Suicide Severity Rating Scale
DSM: Diagnostic and Statistical Manual of Mental Disorders
ECT: Electroconvulsive therapy
MDD: Major Depressive Disorder
IL: Interleukin
MAOI: Monoamine oxidase inhibitor
SSRI: Serotonin Reuptake Inhibitor
MADRS: Montgomery and Asberg Depression Scale
mSv: Milli Sievert
TNF: Tumor Necrosis Factor
TSPO: Translocation protein.
